# Ultrasound Probe Pressure Affects Aortic Wall Stiffness: A Patient-Specific Computational Study in Abdominal Aortic Aneurysms

**DOI:** 10.1007/s10439-024-03608-8

**Published:** 2024-09-04

**Authors:** Marta Irene Bracco, Ali Akbar Karkhaneh Yousefi, Laurence Rouet, Stéphane Avril

**Affiliations:** 1 INSERM, Sainbiose, Mines Saint-Étienne, Saint-Étienne, France; 2 Philips Research Paris, Suresnes, France

**Keywords:** Abdominal aortic aneurysm, Ultrasound probe pressure, Noninvasive mechanical characterization, Finite-element method

## Abstract

**Purpose:**

Ultrasound imaging is key in the management of patients with an abdominal aortic aneurysm (AAA). It was recently shown that the cyclic diameter variations between diastole and systole, which can be quantified with US imaging, increase significantly with the strength of the applied probe pressure on the patient’s abdomen. The goal of this study is to investigate this effect more thoroughly.

**Methods:**

With finite-element modeling, pulsatile blood pressure and probe pressure are simulated in three patient-specific geometries. Two distinct models for the aortic wall were simulated: a nonlinear hyperelastic and a linear elastic model. In addition, varying stiffness was considered for the surrounding tissues. The effect of light, moderate, and firm probe pressure was quantified on the stresses and strains in the aortic wall, and on two in vivo stiffness measures. In addition, the Elasticity Loss Index was proposed to quantify the change in stiffness due to probe pressure.

**Results:**

Firm probe pressure decreased the measured aortic stiffness, and material stiffness was affected only when the wall was modeled as nonlinear, suggesting a shift in the stress–strain curve. In addition, stiffer surrounding tissues and a more elongated aneurysm sac decreased the responsiveness to the probe pressure.

**Conclusion:**

The effect of probe pressure on the AAA wall stiffness was clarified. In particular, the AAA wall nonlinear behavior was found to be of primary importance in determining the probe pressure response. Thus, further work will intend to make use of this novel finding in a clinical context.

## Introduction

Abdominal Aortic Aneurysm (AAA) is a progressive dilation of the aorta resulting in an irreversible bulge-like formation with complex aortic geometries [1, 2]. Despite being asymptomatic during its formation and growth, an untreated AAA can lead to fatal consequences in case of sudden rupture, with massive internal bleeding. More than 20% of post-rupture surgical interventions fail, resulting in a high overall mortality rate [3]. Patients diagnosed with AAA undergo a periodic clinical check to assess the growth rate and rupture risk. Clinical monitoring is typically performed with ultrasound (US) imaging, favored by its non-invasiveness and affordability [4]. Standard clinical guidelines refer to the measurement of the maximum aortic diameter for AAA risk stratification. Specifically, the guidelines set a gender-adjusted threshold above which surgical aneurysm repair is recommended, i.e., 50 mm for women and 55 mm for men. Given the low lateral resolution of abdominal US acquisitions, the standard measurement is the antero-posterior (AP) diameter.

Several clinical studies reported that the maximum diameter criterion alone is not sufficient to predict rupture in all patients, stressing the need for complementary morphological and biomechanical criteria [1]. Biomechanical indexes based on computational stress analysis, namely the peak wall stress and the peak wall rupture index, have been shown to be more sensitive than the gender-adjusted maximum diameter criterion for risk stratification [5].

While these wall stress predictions are widely accepted, accurate non-invasive predictions of aneurysm wall strength are still lacking. As in many other materials, significant changes in the tissue structure precede rupture in the aorta, which also alter its stress–strain relationship. The stress–strain relationship of the aortic wall exhibits a J-shaped curve, with moderate stiffness at low strains (further referred to as *toe* stiffness) and larger stiffness at high strains (further referred to as *heel* stiffness) [6]. Due to the pathological alterations in the tissue during AAA development and progression, the J-shaped curve evolves with the disease [7]. Three consecutive stages were proposed based on biological and mechanical analysis [8]. At Stage 1, the heel stiffness increases significantly, whereas the toe stiffness remains almost constant. At stage 2, the transition from the moderate toe stiffness to large heel stiffness is delayed, mainly because newly deposited collagen fibers are recruited at higher strains. Finally, Stage 3 occurs after significant fibrotic remodeling, yielding very early transitions from moderate to large stiffness.

Given these structural and mechanical changes undergone by the aorta, the in vivo aortic stiffness could be an indicator of AAA progression. A common strategy to estimate aortic stiffness consists in calculating the ratio of the pulsed blood pressure $\Delta P$ to the cyclic diameter variations $\Delta D$, caused by $\Delta P$ in the aortic wall. Strain can be measured globally as $\Delta D / D_\text{dias}$, where $D_\text{dias}$ is the diastolic AP diameter [9], or locally with US tracking [10–13]. However, results obtained so far have not allowed to establish a clear relationship between wall stiffness and AAA growth. While [14] found stiffness to be a poor predictor of AAA growth, [15] found that AAA progression is associated with an increase in aortic stiffness. In addition, wall stiffness and AP diameter were found to be independent predictors of AAA rupture [9].

The variability of results could be partially accounted for by the poor reproducibility of $\Delta D$ measurements. The nonlinear behavior of the aorta [2] may be another significant confounding factor. In a previous study, we observed that the in vivo stiffness decreased when applying a firm probe pressure on the patient’s abdomen, with a responsiveness that varied among patients [16]. To understand how this effect relates to the stiffness transition from toe to heel, and enable more robust stiffness measurements for AAA diagnostics, the mechanics of probe pressure transmission through abdominal soft tissues needs to be elucidated [17, 18]. Accordingly, we report patient-specific finite-element simulations of probe pressure effects on the abdomen and on aortic stiffness. In the following, we describe extensively our modeling approach in the “Materials and Methods” section, then report the obtained results for different patient-specific conditions in the “Results” section, and discuss the potential of these results for AAA US exploration in the “Discussion” section.

## Materials and Methods

**Fig. 1:**
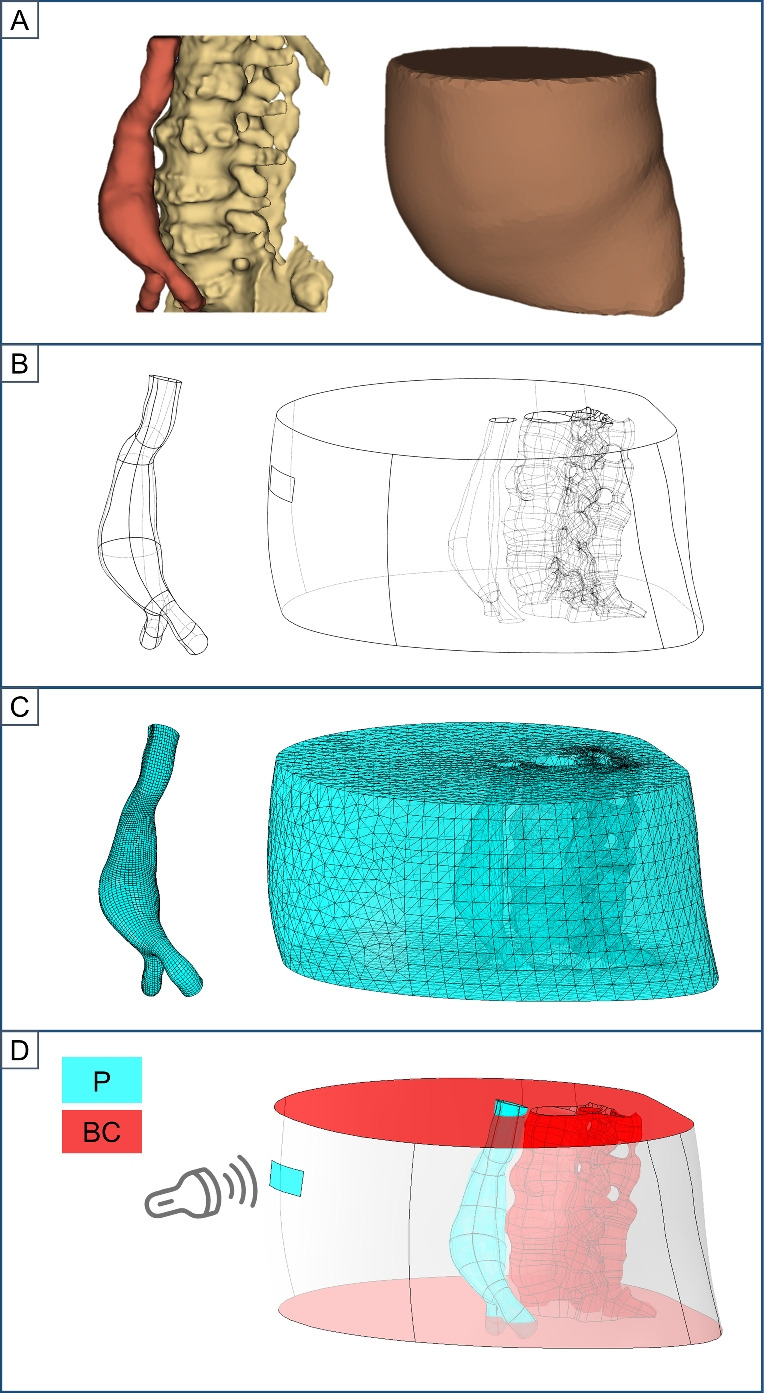
Overview of the simulation workflow. First we segmented patient-specific CT scans (**A**), then defined 3D solid parts (**B**), and generated the finite-element meshes (**C**). Afterwards, we prescribed the pressures (P) and boundary conditions (BC) (**D**), where the pressures were applied to the surfaces depicted in light blue. Specifically, we assigned the pulsed blood pressure inside the aorta, and the probe pressure onto the external surface of the abdomen. The boundary conditions were applied to the surfaces depicted in red. Displacement and rotations were blocked on the spine, while out-of-plane transverse displacements were fixed at both the aortic inlet and outlet

### Data Acquisition

Three patients were recruited for AAA monitoring at the Rigshospitalet Copenhagen. None presented an intra-luminal thrombus. Each patient had a CT scan in their clinical standard of care. The study was approved by the Danish research ethics committee (record number H- 20001116), and each patient signed an informed consent. Imaging parameters slightly differed among patients. Specifically, only one of the scans was acquired with a contrast medium for angiography, referred here as Patient 1. CT scans were stored in Digital Imaging and Communications in Medicine (DICOM) format. Measurements of systolic and diastolic pressures were performed by clinicians with a brachial sphygmomanometer.

### Biomechanical Modeling

We established a pipeline to reconstruct patient-specific finite-element models of the abdomen with a AAA and to simulate the deformations induced by the combination of the pulsed blood pressure and the probe pressure. An overview of the workflow is shown in Fig. 1. In the following, we describe the different steps of this pipeline and then we explain how it was employed to investigate and quantify the effects of surrounding tissues and probe pressure on strain distributions and stiffness estimation during AAA exploration with US.

#### Image Segmentation

The DICOM files were read and processed with the 3D Slicer Software for manual segmentation [19]. Each segmentation was performed by a junior researcher in biomechanics and took around 4 h. The abdominal aorta was segmented from around 6 cm above the aneurysm neck to around 3.5 cm below the iliac bifurcation, to avoid edge effects on the stress analysis in the middle of the AAA. The AAA was segmented by combining gray-level thresholding at 60 Hounsfield unit (HU), islands, and holes filling. Manual refinement was needed, especially for non-contrasted images. The secondary branches up to the renal ones were manually removed. With a similar approach, the spine was segmented between vertebrae S1 and T11 with a 90 HU threshold. Surrounding soft tissues, comprising the abdomen, the internal organs, and the low back muscles, were segmented as a single volume with a 200 HU threshold. We exported final segmentations as a tessellated surface in Standard Triangulation Language (STL) format and smoothed it in Meshmixer (Autodesk, Inc.). The extremities of the aortic branches were cropped with plane cuts perpendicular to the centerlines. An example of segmentation is shown in Fig. 1A, where the aorta is shown in red, the spine in yellow, and the abdominal volume in brown.

#### Model Generation

After segmentation, the aortic centerline and the perpendicular cross-sectional plane located at maximum diameter were computed using the Vascular Modeling Toolkit (VMTK) [20]. Following clinical guidelines for AAA US monitoring, we focused our study on this cross-sectional plane [21]. Segmented surfaces were converted into solid parts with the SpaceClaim software (ANSYS, Inc.) and exported in Standard for the Exchange of Product Data (STEP) format. Then, using the computer-aided design software Fusion 360 (Autodesk, Inc.), the volume of the spine was subtracted from the surrounding tissues volume by intersecting the AAA and the spine, obtaining the soft tissues volume as shown in Fig. 1B.

The probe location on the anterior surface of the surrounding tissues was determined according to the maximum diameter of the AAA. First, VMTK was used to detect the maximum diameter cross-sectional plane. Then, this plane was intersected with the patient’s abdomen (anterior and close to the umbilicus, according to clinical standards). Finally, a rectangular area aligned with the plane was defined. Its dimensions were set to 20 mm $\times$ 70 mm to match with the Philips C5-1 curved array US transducer footprint. The final model comprised the AAA segment and the soft tissues volume, as illustrated in Fig. 1B where the intersection cut with the surrounding tissues is shown on the right hand side.

#### Mesh

The built-in mesh generator of the ABAQUS CAE software (Dassault Systemes, Inc.) was used for the discretization of the solid domains in tetrahedra. To facilitate meshing, the aortic wall was partitioned along the longitudinal direction at the maximum diameter, neck, bifurcation, and branches, and along the circumferential direction. The aorta was meshed with approximately 5500 linear rectangular shells (S4R) with a constant thickness of 2 mm, because local thickness measurement was not possible given the resolution of CT scans. Local circumferential and longitudinal directions were defined for each shell element to assign the collagen fiber orientation , calculated with respect to the circumferential direction.

After performing a mesh sensitivity analysis, it was decided to mesh the soft tissues volume with approximately 200,000 hybrid linear tetrahedral elements (C3D4H).

#### Contact and Boundary Conditions

A plane stress condition was assigned to the aortic wall. Contact with the soft tissues was defined with a surface-to-surface tie constraint, where all the forces are directly transmitted at the interface between the two parts.

A Z-symmetry boundary condition, Z being the direction perpendicular to the transverse plane, was applied to the superior and inferior surfaces of the soft tissues, as illustrated in Fig. 1D, so that only nodal displacements and rotations in the transverse plane are allowed. Due to the tie constraint, the same boundary conditions are transferred to the extremities of the vessel. To simulate the spine constraint, all the nodes at the inner surface of the soft tissues corresponding to the spine cut were clamped.

#### Zero Pressure Configuration

As the segmented geometry was obtained at diastole in a pressurized state, we had to derive the zero pressure (0P) configuration to define the initial load-free geometry needed for further analyses. The iterative procedure to derive 0P was introduced by [22]. Briefly, diastolic pressure was applied to the inner surface of the AAA, and the obtained displacement was reversed to move each initial nodal position inwards. Diastolic pressure was applied again onto the updated geometry, and the procedure was repeated iteratively until obtaining the best agreement possible between the initially segmented geometry and the computed pressurized geometry. Pressure distributions at the inner surface of the AAA were assumed homogeneous.

#### Material Modeling

Two different material models were employed for the aortic wall tissue, either with a linearized [23] or with a nonlinear stress–strain relationship. Specifically, in the linearized case, a linear elastic orthotropic material was assigned to the AAA wall, while in the nonlinear case, the AAA wall was modeled as a nonlinear anisotropic hyperelastic material, following the Holzapfel–Gasser–Ogden (HGO) formulation [6]. The HGO model describes the AAA wall material as a fiber-reinforced composite, where an isotropic matrix component carries the loads at low pressures, and the embedded collagen fibers are engaged as the stress on the wall increases. The soft tissues surrounding the aorta were modeled as an incompressible homogeneous Neo-Hookean material, as reported in [17]. We tested different values for surrounding tissues material constants in order to assess how they affected the obtained aortic stiffness. All material parameters are reported in section “Study Design”

#### Loads

For each of the three patient-specific geometries, we simulated the aortic distention in response to the blood pressure. Patient-specific diastolic and systolic blood pressures were applied to the inner surface of each AAA geometry to simulate a single cycle of pulsed blood pressure.

Moreover, to model the probe pressure, a constant external pressure was applied to the probe surface, as described in Sect. “Model Generation.” For each patient, we applied three different probe pressure conditions: a light probe pressure of 2 kPa (LPP), a moderate probe pressure of 15 kPa (MPP), and a firm probe pressure of 30 kPa (FPP). These values were defined in agreement with previous work on probe pressure [16, 24].

### Mechanical Estimations

The diameter stiffness index, $\beta _\text{diam}$, was derived as follows:

1 $$\begin{aligned} \beta _\text{diam}=\frac{\ln ({P_\text{sys}/P_\text{dias}})}{\Delta D/D_\text{dias}} \end{aligned},$$

where $\Delta D = D_\text{sys}-D_\text{dias}$ is the measured change of AP diameter between diastole and systole [9].

In addition, the circumferential stiffness index, $\beta _\text{circ}$, was derived as follows:

2 $$\begin{aligned} \beta _\text{circ}=\frac{\ln ({P_\text{sys}/P_\text{dias}})}{\Delta C/C_\text{dias}} \end{aligned},$$

where $\Delta C = C_\text{sys}-C_\text{dias}$ is the measured change of circumference between diastole and systole.

#### Elasticity Loss Index

After measuring the AP diameter variations and the circumferential variations in LPP and FPP conditions, two indexes are derived, indicating the loss of elasticity of the blood vessel wall due to the change in probe pressure. The first index is obtained as the ratio between the diameter change in FPP and in LPP conditions. This ratio was named *diameter elasticity loss index* (${ELI}_\text{diam}$), and can be derived as follows:

3 $$\begin{aligned} {ELI}_\text{diam} = \frac{\beta _\text{diam,LPP}}{\beta _\text{diam,FPP}} \end{aligned}.$$

Substituting 2 and 1 in 3, blood pressure ratio (constant) cancels out and ELI can be estimated only with parameters directly derived from US:

4 $$\begin{aligned} {ELI}_\text{diam} = \frac{\Delta D _\text{FPP}}{\Delta D_\text{LPP}} * \frac{D_\text{dias,LPP}}{D_\text{dias,FPP}} \end{aligned}.$$

Similarly, a second index was defined as the ratio between the circumference change in FPP and in LPP conditions, referred to as the *circumferential elasticity loss index* (${ELI}_\text{circ}$), which can be obtained as follows:

5 $$\begin{aligned} {ELI}_\text{circ} = \frac{\Delta C _\text{FPP}}{\Delta C_\text{LPP}} * \frac{C_\text{dias,LPP}}{C_\text{dias,FPP}} \end{aligned}.$$

**Fig. 2:**
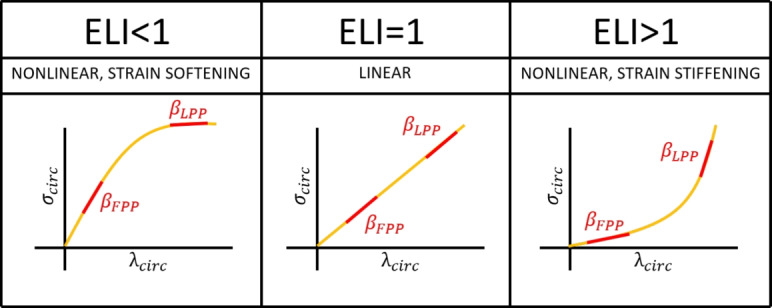
Interpretation of the Elasticity Loss Index (ELI). The ratio between the two tangent stiffness indexes measured at firm (FPP) and light probe pressures (LPP). It is assumed that the circumferential stretch ratio $\lambda _\text{circ}$ is obtained as either the relative diameter or the relative circumference change. In addition, it is also assumed the circumferential stress $\sigma _\text{circ}$ results from the combination of blood pressure and probe pressure. The corresponding material models are specified.

For each simulated set of parameters, $\beta _\text{diam}$, $\beta _\text{circ},$ and ${ELI}_\text{diam}$, ${ELI}_\text{circ}$ were evaluated. The interpretation of ELI is presented in Fig. 2. Here, it is assumed that the circumferential stretch ratio $\lambda _\text{circ}$ can be expressed either as the diameter or the circumference change, and that the circumferential stress $\sigma _\text{circ}$ results from the combination of blood pressure and probe pressure.

### Study Design

The AAA wall material parameters were taken from [25] for the HGO model, which correspond to a healthy aorta from elderly individuals, as reported in Table 1.

**Table 1:** Nonlinear anisotropic material parameters for the AAA wall, from [25]

$C_{10} \text{(kPa)}$	$k_{1} \text{(MPa)}$	$k_{2}$	$\kappa$	$\theta$ ($^{\circ }$)
100.9	4.07	165.55	0.16	48.4

Mechanical parameters: $C_{10}$ = initial stiffness (kPa), $k_{1}$(MPa), and $k_{2}$ (adimensional) = anisotropic stiffness parameters. Structural parameters: $\kappa$ (adimensional) = collagen fibers dispersion coefficient, $\theta$ ($^{\circ }$) = collagen fibers orientation angle w.r.t. circumferential direction

For the corresponding linearized orthotropic model, the parameters were taken from [23], and they are reported in Table 2.

**Table 2:** Linear orthotropic parameters for the AAA wall, from [23]

$E_{\theta }$	$E_\text{z}$	*G*	$\nu$
1.11 MPa	3.58 MPa	4.0 MPa	0.44

$E_{\theta }$ = circumferential elastic modulus, $E_\text{z}$ = longitudinal elastic modulus, *G* = shear modulus, $\nu$ = Poisson’s ratio

These models and sets of parameters, already present in the literature, allowed to compare similar in vivo behavior with different stress–strain relationships.

Finally, three types of surrounding tissues were simulated by varying their shear moduli $\mu$: 20 kPa (stiff) as reported in [17], 10 kPa (medium) and 5 kPa (soft).

The sets of parameters for the 54 simulations are summarized as follows:

$$\begin{aligned}  &   \begin{vmatrix} {\textbf {Patient}}\\ P1 \\ P2 \\ P3 \end{vmatrix} \times \begin{vmatrix} {\textbf {Probe Pressure}}\\ Light \\ Moderate \\ Firm \end{vmatrix} \times \begin{vmatrix} {\textbf {Surrounding Tissues}}\\ Soft \\ Medium\\ Stiff \end{vmatrix} \\  &   \qquad \times \begin{vmatrix} {\textbf {AAA Model}}\\ HGO \\ Linear\\ - \end{vmatrix} = {\textbf {54}} \end{aligned}.$$

## Results

### Patient-Specific Parameters

#### Blood Pressure

Table 3 reports, for each patient, the diastolic and systolic pressure values ($P_\text{diast}$, $P_\text{syst}$) measured by brachial sphygmomanometry. To derive the systolic and diastolic abdominal aortic pressures, $P_\text{dias}$ was decreased by 12% and $P_\text{sys}$ was increased by 5%, as previously reported [26].

**Table 3:** Patient-specific brachial blood pressure values ($P_\text{diast}$, $P_\text{syst}$) (mmHg)

Patient	$P_\text{diast}$ (mmHg)	$P_\text{syst}$ (mmHg)
1	76	138
2	90	153
3	88	144

#### Segmented Geometries

**Fig. 3:**
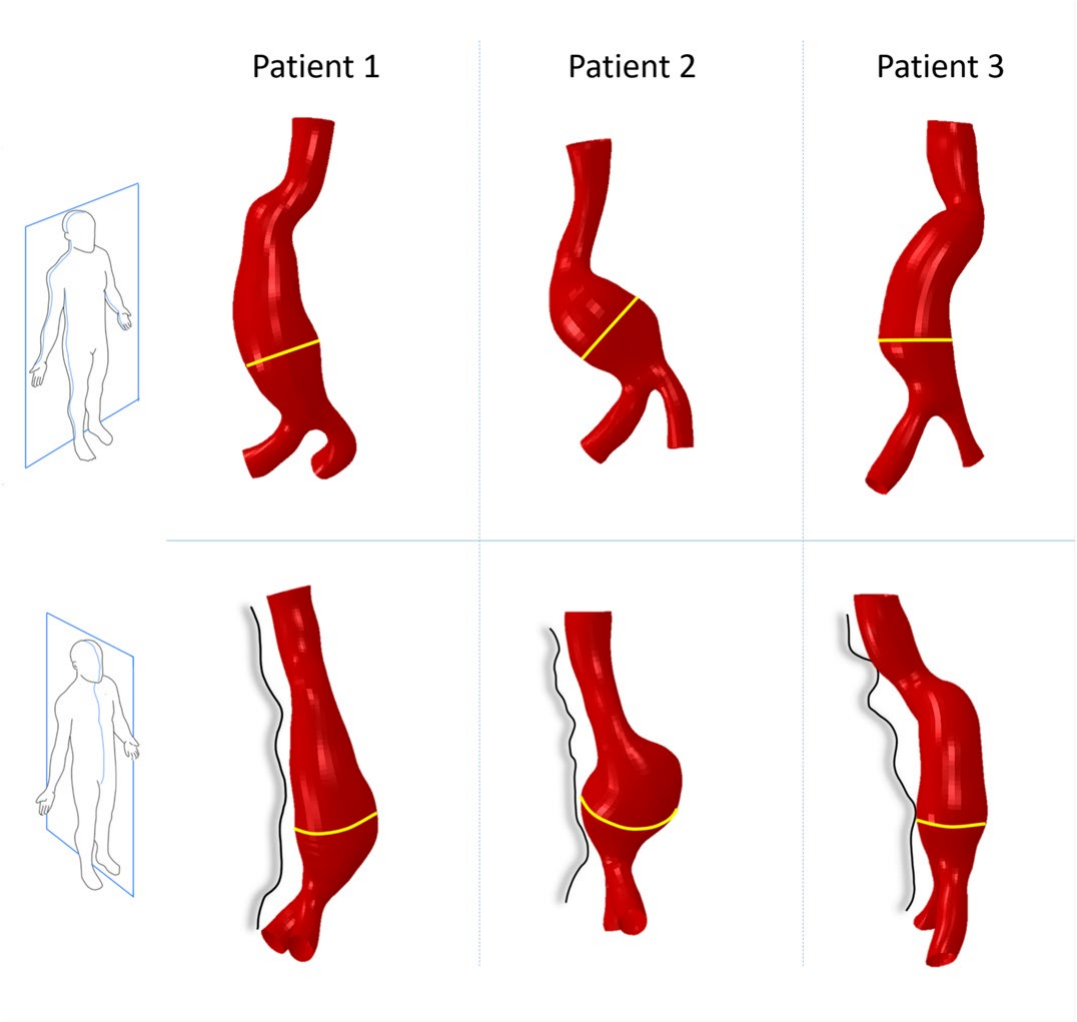
Patient-specific AAA geometries segmented from CT scans. The top row shows the anterior views for Patients 1, 2 and 3. The corresponding lateral views are in the bottom row, together with a delineation of the vertebral spine. The maximum diameter sections are shown in yellow

The three patient-specific AAA geometries obtained by segmenting CT scans are shown in Fig. 3. In the lateral views, the anterior contour of the vertebral spine near the AAA, can be appreciated. Patients 1 and 3 show an elongated aneurysm sac compared to Patient 2, which presents a more spherical shape. In particular, in patient 3 the maximum diameter cross section is not as distinct as in the other cases. Patients 2 and 3 also present twisted geometry, with a pronounced antero-posterior (Patient 3) and lateral (Patient 2) misalignment between the neck and the bifurcation.

### Biomechanical Modeling: Simulation Results

**Fig. 4:**
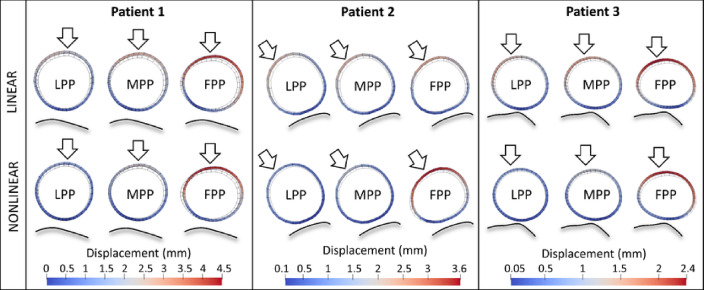
Simulation results plotted at the maximum diameter cross section for each patient-specific geometry. The diastolic (dotted line) and systolic (solid line) geometries are shown together. Color maps of total displacement from diastole to systole are displayed. Probe pressure increases from left to right. Results are shown for linear (top) and nonlinear HGO (bottom) AAA material assumptions. Color scales are reported below each patient. The spine contours are delineated (shaded black lines), as well as the probe pressure direction (white arrows). The wall thickness is depicted for visualization purposes.

**Fig. 5:**
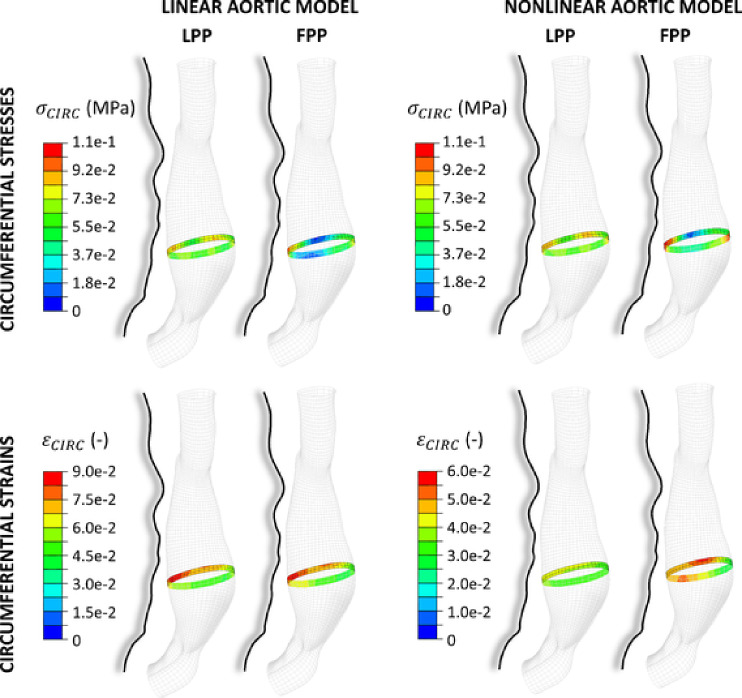
Simulation results are shown for Patient 1, with a linear (left) or a nonlinear (right) aortic material model, and soft surrounding tissues. The displayed color maps represent the circumferential stresses ($\sigma _\text{circ}$, top), and strains ($\varepsilon _\text{circ}$, bottom) around the scanning plane. $\sigma _\text{circ}$ result from diastolic blood pressure and probe pressure combined. $\varepsilon _\text{circ}$ result from the transition from diastolic to systolic blood pressure, at constant probe pressure. Light (LPP) and firm (FPP) probe pressure results are presented. The scales are reported on the left hand side for each case. The spine contours are delineated (shaded black lines).

Figure 4 presents the geometry and in-plane wall displacements within the maximum diameter cross section, from diastole to systole, for the three patients, while increasing probe pressure is applied, from the left (LPP) to the right (FPP). The effect of probe pressure on the cross-sectional geometry can be appreciated. The results are reported both for the simulations with a linear (top) and with a nonlinear (bottom) aortic material assumption. Figure 5 depicts the stresses (top) and strains (bottom) in the circumferential direction, within the surface of the wall adjacent to the cross section, for Patient 1. The orientation of the AAA is lateral, with the spine on the left and probe on the right, as in Fig. 3 (bottom). The obtained stresses are caused by the combined effect of diastolic blood pressure and varying probe pressure values (LPP and FPP), while the strains are the cyclic strains induced by the cardiac cycle. The same plots are illustrated both for the linear (left) and nonlinear (right) aortic material model assumptions. It is possible to see that the stress distributions and values change in both cases. When going from LPP to FPP, the lateral walls are compressed, and the value of stress tends towards zero, while the tension in the anterior and posterior wall increases up to 110 kPa. The cyclic strains present different responses to probe pressure depending on the aortic model assumption. In the linear case, the strain distribution is affected similarly by LPP and FPP. Conversely, in the nonlinear case, both the strain distributions and values appear to be significantly more affected by FPP. This is particularly evident on the lateral walls, where the strain increases from around 4% up to 6%, while remaining at the same level on the anterior wall.

**Fig. 6:**
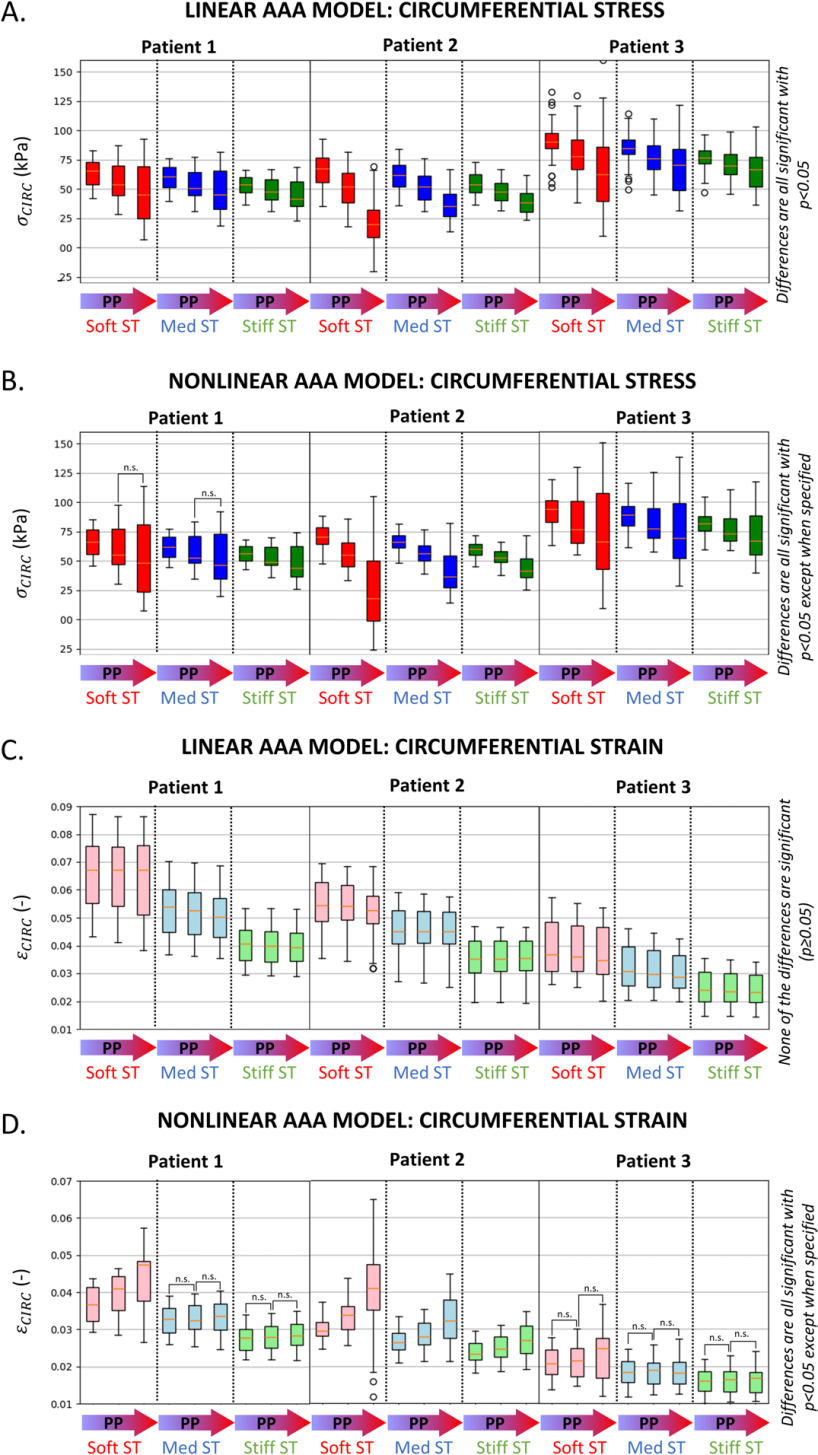
The effect of the probe pressure on the wall circumferential stresses ($\sigma _\text{circ}$, **A, B**) and circumferential strains ($\varepsilon _\text{circ}$, **C, D**) is shown, assuming either a linear (**A, C**) or a nonlinear (**B, D**) aortic model. **A** and **B** box plots report the stresses $\sigma _\text{circ}$ resulting from the combined effects of blood pressure and probe pressure in the elements (n=82±4) within the maximum cross section, as shown in Fig. 5. **C** and **D** box plots summarize $\varepsilon _\text{circ}$ in the same area due to the transition from diastolic to systolic blood pressure, at constant probe pressure. Growing external probe pressure values are considered: light (LPP), moderate (MPP), and firm (FPP). Surrounding tissues (ST) are assumed soft (red), medium (blue), or stiff (green). Significance (*t* test, *p* < 0.05) is indicated.

   These results are generalized for all patients in Fig. 6. The circumferential stresses experienced by the AAA around the maximum diameter cross section are reported assuming linear (Fig. 6A) and nonlinear (Fig. 6B) AAA models, showing the effect of probe pressure (LPP, MPP, FPP) and of surrounding tissues stiffness (red, blue, green). The three patients behave similarly in all simulations. The overall median stress is decreased within the cross section when transitioning from LPP to FPP, while it becomes more heterogeneous. As the surrounding tissues stiffen, the heterogeneity of stresses decreases.

The circumferential strains due to the blood pressure (or cyclic strains) are reported in Fig. 6C for the linear aortic assumption cases and in Fig. 6D for the nonlinear cases. Increasing the probe pressure in the linear case did not lead to a substantial change in the cyclic strains. Conversely, in the nonlinear case, both median and heterogeneity of the cyclic strains increase with the probe pressure, especially in Patients 1 and 2. In addition, the overall median strains decrease with increasing surrounding tissues stiffness in all cases.

### Mechanical Estimations

#### Stiffness

**Fig. 7:**
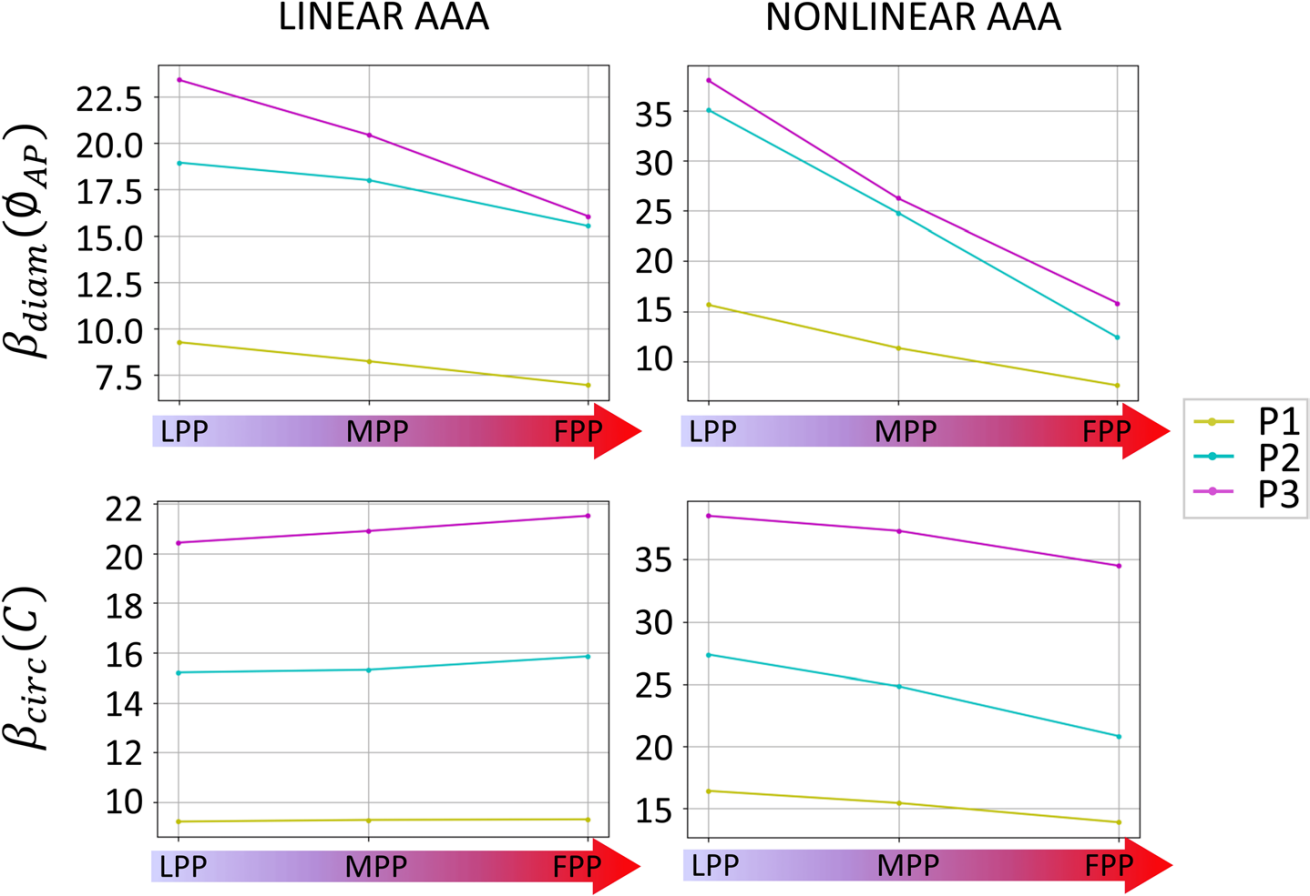
Stiffness indexes $\beta _\text{diam}$ (top) and $\beta _\text{circ}$ (bottom). Each color represents one patient (P1, P2, P3). The values of stiffness are reported for each probe pressure condition: light (LPP), moderate (LPP), and firm probe pressure (FPP). Results are reported for soft surrounding tissues. Results were obtained using a linear aortic material model (left) and a nonlinear aortic material model (right). A decrease in measured stiffness with probe pressure can be observed consistently in all cases except for $\beta _\text{circ}$ with linear model.

Figure 7 presents the stiffness values calculated from the simulation results. Each box plot summarizes the values obtained from the three patients. The probe pressure effect can be appreciated together with the effect of increasing surrounding tissues stiffness. $\beta _\text{diam}$, calculated starting from the AP diameter changes, shows an overall decrease due to probe pressure. Conversely, $\beta _\text{circ}$, calculated starting from the circumference changes, remains stable as the probe pressure is increased when assuming a linear aortic model, while it decreases when assuming a nonlinear material. In all cases, the AAA appears stiffer at an increased ST stiffness.

#### Elasticity loss index

**Fig. 8:**
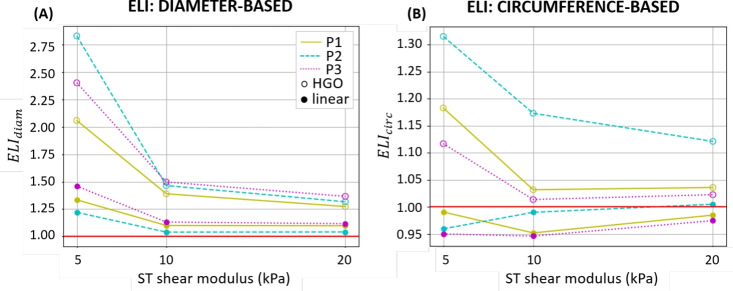
Diameter and circumference elasticity loss indexes (${ELI}_\text{diam}$, ${ELI}_\text{circ}$) calculated as the ratio of the (A) relative diameter changes or (B) relative circumference changes, measured for firm and light probe pressure conditions, plotted against the surrounding tissues (ST) shear moduli: 5 kPa, 10 kPa, and 20 kPa corresponding to soft, medium, and stiff ST, respectively. Each line refers to a different patient-specific geometry: solid, yellow line for Patient 1 (P1), dashed, cyan line for Patient 2 (P2), and dotted, magenta line for Patient 3 (P3). Results are reported for a linearized material model (filled circle) and a nonlinear material model (empty circle). Horizontal red axis indicates where the *ELIs* are equal to 1. The *ELI* is positive and decreases with increased ST stiffness in all cases except when a linear model is assumed for the aortic wall and ${ELI}_\text{circ}$ is measured.

Figure 8 shows the calculated indexes ${ELI}_\text{diam}$ and ${ELI}_\text{circ}$ plotted against the surrounding tissues stiffness. The maximum values of ${ELI}_\text{diam}$ and ${ELI}_\text{circ}$ are found using the nonlinear material assumption for the AAA wall and soft surrounding tissues ($\mu$ = 5 kPa). These values decrease when stiff surrounding tissues are simulated ($\mu$ = 20 kPa), although the biggest decrease in ELI is found when going from soft to medium surrounding tissues. Both ELIs decrease when a linear model for the AAA is used. Specifically, ${ELI}_\text{diam}$ follows the same tendency as in the nonlinear case: it decreases as the surrounding tissues stiffness increases. Conversely, ${ELI}_\text{circ}$ shows a slight increase.

## Discussion

Previous findings showed that US probe pressure can affect in vivo measurements of the AAA wall stiffness. To better understand this effect and its implications, we developed patient-specific finite-element models of AAA embedded in the abdomen under US probe compression. More specifically, we investigated the effects of material nonlinearity, patient-specific AAA geometry, and surrounding tissue stiffness on the circumferential stresses and strains experienced by the AAA. The outputs of our finite-element simulations were the diameter stiffness index, denoted $\beta _\text{diam}$, which are currently available in clinical settings, and the circumference stiffness index, denoted $\beta _\text{circ}$. As these stiffness predictions varied with the applied probe pressure, we derived the ELI ratio, which is the ratio between the stiffness measured at FPP and the one measured at LPP. We propose this ratio as an indicator of wall tissue nonlinearity, as it is related to the transition from toe to heel stiffness in the J-shaped stress–strain relationship.

In order to demonstrate that the variations of stiffness when varying the probe pressure are related to the material nonlinearity, we also computed the ELI ratio for the same geometric models but by turning the nonlinear hyperelastic model of the AAA into a linear elastic model. It was confirmed that a firm probe pressure makes the in vivo stiffness decrease. The stiffness decrease could be explained by the shift of in vivo stresses and strains towards the more compliant toe region of the J-shaped curve of the material behavior. When we modeled the AAA with a linear behavior, this shift occurred as well, but the stiffness remained unchanged by definition. Nevertheless, we also found that, unlike the $\beta _\text{circ}$ stiffness, the $\beta _\text{diam}$ stiffness was decreased by the probe pressure whether the material was modeled as linear or nonlinear. Such observation suggests that the $\beta _\text{diam}$ stiffness is not only related to the material stiffness, but also to a structural stiffness depending on the shape of the AAA. Interestingly, $\beta _\text{circ}$ only decreased when the nonlinear material model was used, indicating that $\beta _\text{circ}$ is only related to the material stiffness. As a consequence, the ratio ${ELI}_\text{circ}$ can be used as an indicator of AAA material nonlinearity.

In addition, the effect of the surrounding tissues was studied. ${ELI}_\text{circ}$ was lower than 1 when a linear model for the AAA was assumed, meaning that the ability of the AAA to expand under blood pressure is reduced due to the surrounding tissues becoming stiffer as they are compressed by the probe. Given that surrounding tissues behave as a neo-Hookean hyperelastic solid, their stress–strain relationship in compression may have a reversed J-shape. Thus, the more they are compressed by the probe pressure, the stiffer they become, which explains why ${ELI}_\text{circ}$ in the linear case does not equal 1.

Increasing the shear modulus of the surrounding tissues allowed to simulate these tissues with a steeper stress–strain curve. In general, the stiffer the simulated surrounding tissues, the less intense the response to probe pressure. However, the role of surrounding tissues does not seem prevalent enough to invert the in vivo response. Therefore, the two groups found in our previous study, one responsive and one non-responsive, could be also partially explained by the patient-specific composition of the surrounding tissues [16].

Finally, from the comparisons presented in Fig. 6, it is possible to observe the impact of the native AAA geometries on the patient-specific responses. Namely, the strains experienced by the AAA wall in Patient 3 seemed less affected by probe pressure than in the other two cases. Such behavior could be due to the more elongated shape of the AAA, which might increase the constraint exerted by the surrounding tissues, although more patient-specific geometries are needed to confirm this finding.

### Limitations

The simulation approach presented in this study has some limitations. First, axial pre-stretch and circumferential residual stresses were neglected in the simulation to decrease the computational complexity. They are usually not as significant in AAA as they may be in healthy abdominal aortas, but they may still induce some change in the stress–strain curve and these effects should be investigated computationally in the future by running sensitivity analyses.

Second, the wall thickness could not be assessed from the CT scans; thus, it was assumed homogeneous and was set to 2 mm [27]. It has been previously shown that wall thickness is patient-specific and has a significant effect on AAA pathological developments [28]. In addition, aortic wall thickness distribution was found to be non-uniform and to have an effect on mechanics [29]. Therefore, efforts should be put into the development of methods to extract local, patient-specific thickness values. Finally, the AAA was modeled using the generic constitutive parameters of aortas from elderly individuals, simply because the associated linearized parameters were available. Given that the stress–strain relationship in AAA becomes even more nonlinear [8], the present results are likely to be confirmed and even more pronounced with the patient-specific material properties of AAA.

In addition, we performed the current study on AAA patients without intra-luminal thrombus and calcification. Calcifications are known sources of local stress concentrations, while the intra-luminal thrombus can affect the overall stress distribution [30]. In a future study, more AAA patients, including patients presenting these two features, should be included.

Finally, the assumption of homogeneous surrounding tissues remains an idealized model for the complex anatomy of the abdomen. In a previous study, surrounding tissues were modeled with four spring elements [18]. Subdividing the surrounding tissues domain into lateral and anterior portions with different material properties could help better understand their influence on AAA mechanics.

### Conclusion

In this work, we explored, for the first time to our best knowledge, the patient-specific effects of probe pressure on AAA biomechanics. We found that, due to AAA material nonlinearity, the apparent aortic stiffness differs when applying a firm probe pressure and when applying a low probe pressure. The circumferential strains induced in the AAA wall by the intra-luminal blood pressure are reduced under a firm probe pressure. Given the convex shape of the stress–strain relation, the reduction of strain results in a reduction of stiffness under a firm probe pressure. Therefore, the magnitude of this reduction, quantified through the ${ELI}_\text{circ}$ ratio, could be used to estimate the curvature of the stress–strain relation. A large ${ELI}_\text{circ}$ ratio indicates a J-shaped stress–strain relation with a large curvature, whereas a low ${ELI}_\text{circ}$ ratio indicates a more linear stress–strain relation. As the progression of AAA was recently related to the shape of AAA stress–strain relation [8], we will investigate more thoroughly the possible correlations between ${ELI}_\text{circ}$ and biological alterations occurring in AAA to ultimately propose robust aortic stiffness measures in the decision-making process of AAA clinical management.
